# Indicators of satisfaction in clickers-aided EFL class

**DOI:** 10.3389/fpsyg.2015.00587

**Published:** 2015-05-06

**Authors:** Zhonggen Yu

**Affiliations:** ^1^School of Foreign Languages, Hohai UniversityNanjing, China; ^2^School of English, Zhejiang Yuexiu University of Foreign LanguagesShaoxing, China

**Keywords:** clickers, indicators of satisfaction, interaction, self-regulation, self-efficacy

## Abstract

How to identify whether students are satisfied with clickers-aided EFL class might be largely a mystery for most researchers since satisfaction is deeply hidden in human psychology which is subtle and intangible. This study, by using bivariate correlation analysis and structural equation modeling, survey scales claimed both valid and internally consistent, and data collected from randomly selected 227 participants, explored the indicators of satisfaction in clickers-aided EFL class, together with gender differences in the indicators. It was concluded that satisfaction was positively correlated with interaction, self-efficacy and self-regulation in clickers-aided EFL class without statistically significant gender differences. Furthermore, interaction, self-efficacy and self-regulation were mutually and significantly correlated. Although indicators of satisfaction might not be limited to these three factors, the findings should be helpful to future researchers who desire to determine whether users are satisfied with the polling technology. Then teachers could decide what teaching style and contents should be adopted. In order to satisfy users of clickers, future lecturing might be designed to promote peer interaction, self-efficacy and self-regulation.

## Introduction

A number of researchers and institutions acknowledged the value of technology use in teaching and learning (e.g., White et al., 2011; Yu and Liu, 2014; Yu et al., 2014). Advanced technologies have been proved to provide learners and teachers with a convenient medium in learning and instruction. It has been widely acknowledged that technology can enhance the process of learning, by accelerating learning while reducing cognitive loads (Yu et al., 2014).

Personal hand-held responders, often referred to as “clickers” or “clicker” are one of the latest technologies used for learning and teaching (Duncan, 2005; Beatty, 2013). Clickers are a kind of technology easily adopted in education (Bruff, 2009). Clickers are also called a Classroom Communication System, Student Response System, or Audience Response Technology, referring to inquiry-based teaching strategies coupled with clicker technology system, a computer technology that enables instructors to ask questions and require students to respond using hand-held devices (clickers). The questions and answers summarizing student responses can be displayed simultaneously on the multimedia projector (Han and Finkelstein, 2013). Clickers with a long history are widely used in many educational institutions in the United States. For example, at the University of Colorado, in 19 departments and 80 courses, over 10,000 clickers were applied during the spring semester 2007 (Han and Finkelstein, 2013). It is obvious that clickers have been widely used in education, which provides a convenient platform for teaching and research. Nevertheless, very few studies explored this field. This study, attempting to identify the indicators of satisfaction in clickers-aided EFL learning, is therefore worthwhile.

## Literature review

### Application of clickers

Some studies showed that clicker system was used earlier in Taiwan. As early as 1995, National Central University began to study how to integrate class feedback with technologies. In 2000, under the leadership of National Central University there were 15 primary schools taking part in the study and started to promote clicker use in local government which was called “Using a clicker system in class.” In 2002, clicker system was widely used among 1000 experimental classes in 150 primary schools in Taiwan. In 2004, Taiwan government set up more than 2000 experimental classes. In Mainland China, interactive teaching and learning was under investigation conducted by research departments assisted by clicker system (Bojinova and Oigara, 2011). In Mainland China there are merely a few studies on use of clicker system, mainly focusing on its effectiveness in educational institutions (e.g., Huang, 2010; Xie et al., 2013). Studies, however, are still sparse in colleges and universities. The purchase and application of clickers in educational institutions remain an awkward issue to be addressed. Studies on use of clickers in China mainly focus on academic achievements and course design, while very few explored use of clickers in terms of satisfaction. This study will explore the indicators of satisfaction in use of clickers so as to suggest that satisfaction of learners and teachers be taken into consideration in future application of clickers in China. This study will also be helpful to future clickers-aided course design.

Although clicker system is relatively less used in China, it has been in use for more than a decade in North America. There have been more than 700 colleges including Harvard University, North-western University, Washington University and Ohio State University, together with some primary and secondary schools where clicker system is used in class.

In America, use of clickers has undergone great innovations since they were born. Some studies showed that clicker system was used in universities in USA as early as 1960s (Judson and Sawada, 2002). During that time, clicker system could not be widely used because of the limitation of technology of wired transference. Until the 1980s, there appeared a wireless clicker device, i.e., an infrared remote control, but the size was too large to hold in hand. After the 1990s, the online version of clicker system began to emerge aided with the popular Internet in Americans' daily life. And there was no charge for the software. Students could directly download the software they needed from the Internet. Clicker technology stepped into a more mature and reliable stage. Clicker system could be used in not only traditional class but also online learning. Other electronic devices were also used in clicker system such as PDA, and mobile phone, etc. (Fang, 2009). With the development of technology, clicker system has recently been widely used in USA and become one of the latest pedagogical technologies.

### Design of clickers-based learning and instruction

Some studies were dedicated to designs and the corresponding effectiveness of clickers-based learning and instruction, the majority of which explored clickers-based pedagogic approach (Jones et al., 2012), design of clickers-aided questions (Hogan and Cernusca, 2013), and means of clickers-aided questions presentation (Perez et al., 2010). Findings from these studies were not in agreement. No statistically significant differences were found in learning outcomes by using peer instruction and individual clicker questions (Rush et al., 2013), which was in contrast to the argument of Smith et al. (2011) who concluded that peer instruction was more closely related to overall learning outcomes than any other clickers-based methods.

In addition, formative and summative assessment and feedback methods led to different findings. Awarding merits for clicker answers would possibly result in cheating (White et al., 2011), which was echoed by Han and Finkelstein (2013) who argued that use of formative feedback could increase student engagement and participation, but it was not true of summative feedback. However, clicker answers could also contribute to participation marks instead of right answers (FitzPatrick et al., 2011). Therefore, it is worth further studies on how awarding merits for clicker answers could impact learning outcomes (White et al., 2011).

### Satisfaction of use of clickers

Use of clickers, in upper-level, average-level, and lower-level classes, received increasing satisfaction. Studies on clicker use in terms of acceptance showed that most students were willing and voluntary to use clicker technology (McKeachie, 1990). The majority of students and lecturers highly valued the use of technology. Lecturers in both basic and advanced psychology courses favored the use of clickers in that the technology effectively incorporated clicker technology with student involvement and student satisfaction in the classroom. It was also found through survey items that clickers were fun to use and receiving credit merely for active response made students more likely to use clicker technology (Smith, 1977).

### Correlation between interaction, self-efficacy, self-regulation, and satisfaction

The framework of this study is to identify whether interaction, self-efficacy and self-regulation can function as indicators of learner satisfaction. Interaction was deemed as an important indicator of learner satisfaction (Bolliger and Martindale, 2004; Bray et al., 2008; Ali and Ahmad, 2011). Öncü and Özdilek (2013) explored undergraduate students' satisfaction through learning interactively with peers. Students from two different departments were grouped into a collective class activity to perceive whether different levels of satisfaction were experienced through interacting with peers. Analysis through data sourcing from a sample of 47 Science Education and 72 Computer Education and Instructional Technology majors demonstrated that both majors were highly and equally satisfied through the interaction. Both learner-learner and learner-instructor interactions were significant indicators of student satisfaction, and learner-instructor interaction was found to be the strongest indicator of student satisfaction (Kuo et al., 2014a). Three types of interaction predicting satisfaction were identified, of which learner–content interaction was the strongest indicator of learner satisfaction when involving less collaborative activities, and those with extroverted personality more likely interacted with peers and teachers, leading to more satisfaction than the introverted learners (Kuo et al., 2014b).

Self-efficacy indicates learner beliefs, confidence, and expectations in fulfilling a specific task (Bandura, 1977), which can exert an important influence on motivation and learning outcomes. An increasing number of studies have regarded self-efficacy as an indicator of success in technology assisted education (Artino and Anthony, 2007; Liang and Tsai, 2008), as well as satisfaction. Çakar (2012) examined the relationship between self-efficacy and satisfaction of young adult learners through cross-sectional methods, with the data collected from randomly sampled young adults who were pursuing bachelor degrees and attending the Celal Bayar University Pedagogical Formation Program. The research instruments were General Self–Efficacy Scale and The Satisfaction with Life Scale. Self-efficacy of learning were found an important indicator of satisfaction (48%, *p* = 0.05). It was therefore indicated that raising self-efficacy could improve satisfaction level. However, it was also argued that self-efficacy was not significantly related to satisfaction (Kuo et al., 2014b).

Self-regulation is defined as the psychological factor to indicate the degree that students metacognitively, motivationally, and behaviorally participate in learning activities (Zimmerman and Schunk, 1989). Metacognitive ability implies learners' ability to establish plans, schedules, or goals in order to achieve learning goals. Motivational processes require learners to be self-motivated and voluntary to pursue success in the learning task (Moller and Huett, 2012). Self-regulation combines intrinsic motivation with internalization of extrinsic motivation, which might facilitate satisfaction of learning (Deci and Ryan, 1996; Bembenutty and White, 2013). Self-regulation also involves students managing their own learning, setting goals and subgoals, monitoring progress toward those goals, and rewarding themselves (or otherwise managing their motivation). Nevertheless, merely self-regulation might not realize satisfaction especially for achieving students. Academic achievements might be more important an indicator of satisfaction although self-regulation was positively related to learning outcomes (Cheng and Chau, 2013).

Use of clickers would increase self-regulation to some extent. In the clickers-aided class, students are required to poll immediately after the clicker questions are raised. Each of polling will be projected to the large screen exposed to the whole class. Those who did not poll will be spotted out by the lecturer, which will be recorded as a deficit in the final grade. Student attendance and in-class performance can also be easily reviewed through this polling technology, which will examine students' preparation before class as well. In order to poll in class and keep pace with the progress, students have to cultivate self-regulation under this external control.

Factors to indicate satisfaction are deemed important since teachers tend to feel difficult to determine students' feelings about the use of clickers. Although some studies demonstrated that interaction, self-efficacy, and self-regulation were possibly able to indicate whether learning and teaching were satisfactory, contradictory results still coexisted (e.g., Cheng and Chau, 2013; Kuo et al., 2014b), among which gender differences were an everlasting issue. In case indicators of satisfaction are determined, teachers will be able to satisfy students through meeting the indicators. Course design will be more explicitly guided toward the improvement on indicators of satisfaction. Students will be most likely satisfied by the satisfaction-oriented teaching. This will possibly increase the effectiveness and efficiency of learning and teaching, and finally improve learning outcomes. This study is therefore meaningful and significant.

### Gender differences

Contradictory findings also existed in gender differences in technology aided learning. Females showed stronger capacities of adapting to leaning tasks than boys (Ghazvini and Khajehpour, 2011), which failed to explain that technology aided learning was more satisfactory to males than to females. However, females paid more attention to learning plans, and had more various interactive activities than males (González-Gómez et al., 2012). Females were found to have more technology assisted self-efficacy than males. Males, more exploration-oriented technology users, used educational technology more frequently than females who were more communication-oriented technology users (Tsai and Tsai, 2010).

Nonetheless, gender differences in indicators of satisfaction in clickers-aided EFL have been hardly explored since clickers were put into use in education. By contrast, other learning outcomes such as learning attitude and achievement were well explored (e.g., Tandogan and Orhan, 2007; Kenneth, 2011; Narmadha and Chamundeswari, 2013; Yu and Liu, 2014; Faris, unpublished project paper). Therefore, the focus in this study will be on gender differences in indicators of satisfaction. Consequently, this study, aiming to identify whether interaction, self-regulation, and self-efficacy are indicators of satisfaction for both males and females, seems meaningful and necessary. Moreover, the relationships between these variables will also be identified in order to account for the indicators of satisfaction. The proposed research question in this study is: will interaction, self-regulation, and self-efficacy indicate satisfaction for both male and female learners? Correspondingly, six hypotheses are raised: (1) H1: for males, interaction is an indicator of satisfaction; (2) H2: for males, self-regulation is an indicator of satisfaction; (3) H3: for males, self-efficacy is an indicator of satisfaction; (4) H4: for females, interaction is an indicator of satisfaction; (5) H5: for females, self-regulation is an indicator of satisfaction; (6) H6: for females, self-efficacy is an indicator of satisfaction; (7) H7: self-regulation, interaction, self-efficacy and satisfaction are mutually correlated.

## Methods

The research lacks consent because the data were analyzed anonymously. The research has been approved by the authors' institutional review board-School of Foreign Languages of Hohai University, which waived the need for written informed consent from the participants. All experiments conform to the relevant regulatory standards. The clinical trial was registered in a public trial registry. A select agent or toxin was not used in the experiments in the manuscript. This manuscript does not contain a National Science Advisory Board for Biosecurity (NSABB)-defined experiment of concern.

### Participants

Participants (*N* = 227, Male = 115, Female = 112) were randomly selected from those who pursued their bachelor degrees in a university in China. They were aged between 22 and 25 years old (*M* = 23.42, *SD* = 1.25). They were majored in various disciplines, ranging from arts, humanities, science to engineering. They all received the College English education aided with clicker teaching system for no less than 1 academic year, thus familiar with use of clickers. To conveniently observe student performance and to enhance the reliability of findings, all the recruited participants, across different classes, were taught based on different teaching schedules by different English lecturers who possessed rich experience in clickers-aided teaching and received specialized training. They were informed of the research objectives and voluntary to join the study. In the 1 year's education aided with clickers, they were asked to anonymously poll using the technology, interact with their peers, and the teacher decided the teaching progress based on the scenario projected to the large computer screen. After class, clickers were collected by technicians for maintenance in order to effectively function next time when in use.

### Research instruments

The instrument in this study is mainly a survey including questions on demographics, indicator variables and the outcome variable of student satisfaction (Please see the Supplementary Material), followed by a 5-Likert scale, i.e., *I strongly disagree, I disagree, I don't know, I agree, I strongly agree*, contributing to one to five points respectively for each choice.

The self-efficacy scale, developed by Eastin and LaRose (2000), involves an overall measure related to general clicker use, with items regarding the extent to which participants feel confident in using clickers to discuss with peers, answering questions by clicking the buttons, evaluating their responses and summarizing the results. Scores from the Internet self-efficacy subscale were found to be valid and reliable in previous work (self-efficacy: α = 0.93). Sample questions are “I feel confident understanding terms/words relating to software and hardware of clickers” and“I feel confident learning advanced skills through clickers.”

The scales developed by Kuo et al. (2009) were used to identify the degrees of interaction and satisfaction in blended learning environment. Sample questions are: “Clickers-aided EFL class facilitates feedbacks from peers” and “Clickers-aided EFL class presents an easy access to frequently asked questions” to identify degree of interaction, and “I am well satisfied with clickers-aided EFL class as a whole” and “Clickers-aided EFL class helps improve the satisfaction of learning” to identify satisfaction levels. This scale was claimed reliable (Kuo et al., 2009). The scale items were adapted to the classroom environment assisted with clicker teaching system. To assess the content validity of the scale, seven professors who were expert in statistics calculated the ratio, which involved two-rounds of rating. After this, some items were removed and adjusted based on the suggestions of the experts. A pilot study with 111 respondents was conducted in summer 2009 (Kuo et al., 2013) in order to identify the reliability and the content validity information for interaction, revealing that both reliability and validity reached a satisfactory level (learner–learner interaction: α = 0.99; leaner–instructor interaction: α = 0.88; learner–content interaction: α = 0.92; satisfaction scales: α = 0.93).

The self-regulation scale was adapted from the Metacognitive self-regulation subscale—Control of Learning Beliefs designed by Pintrich et al. (1993), which determined the use of planning, monitoring, and regulating strategies in learning. Scores from self-regulation subscales were found to be satisfactorily valid and reliable in previous studies (self-regulation: α = 0.79). Sample questions are “If I study in appropriate ways, then I will be able to learn the material in this course” and “It is my own fault if I don't learn the material in this course.”

### Procedure

The researcher contacted the course lecturer and informed him of the aim of the study in order to obtain support from him. Under the lecturer's help, the researcher was then familiar with the number, majors, teaching style and other related information. Then the researcher determined the venue and the time and randomly selected the voluntary participants. After recruiting the participants in several classrooms, they were required to fill out the survey under the guide and inspection of the researcher and instructors. All the data collected from the survey were entered into SPSS 13.0 and Amos for further analysis. The clickers-aided EFL instruction will be introduced below.

### A general picture of EFL instruction with clickers

The significant advantages of clickers are anonymous polling and peer discussion. Through anonymous polling, students are activated to make daring choices regardless of the risk making wrong or ridiculous choices. Students' self-regulation is therefore enhanced. On the other hand, peer discussion widens student horizons, enhances the learning atmosphere, bridges the learning gap between individuals, enhances students' self-efficacy, and promotes their interaction.

In the clickers-aided EFL class, the lecturer designed various clicker questions according to language points and course requirements, which were systematically put forward in class. Students were required to answer questions through anonymously clicking the corresponding buttons, whose results would be instantly shown on the large screen in the form of histograms. The histograms contained various kinds of information involving the percentage of correct and incorrect answers, distributions of answers, the number of participants, participant IDs (only revealed to the lecturer), time and venue of polling, and name of the course, etc. Provided the majority of answers were correct, the lecturer would progress to the next topic with a reminder of those making incorrect choices. If the minority of answers were correct, students would be required to join peer discussion to interact with each other and exchange ideas. The lecturer would further explain the question and related knowledge in detail before students polled once more. The lecturer would not move on to the next topic until most students understood and made the right choice. If nearly half students made the right choice and half made wrong, peer discussion would be carried out and the lecturer would then focus on the differences of right and wrong choices until students could fully perceive the knowledge.

Besides, to activate students and promote interaction, the teachers also designed other learning activities such as small group projects, and questioning. In the whole process, participants polled anonymously, and the lecturer knew their IDs in order to encourage student attendance. Student participation in polling, peer discussion and other learning activities would be an important component of their final grade, which aimed to promote student attendance and participation.

## Results

The collected data were entered into and analyzed through SPSS 13.0 to identify the internal consistency and the correlation between different variables. Cronbach's alpha was used to measure the internal consistency of items in each scale. Nonparametric Spearman rank correlations were used to test for relationships between self-efficacy, interaction, self-regulation and satisfaction.

### The bivariate correlations

The Bivariate Correlations procedure computes Spearman's rho with its significance levels because the data were not normally distributed judged from the skewness and Kurtosis shown in Table 1. Correlations measure how variables are related. In this study, through correlation measures, the correlation between three variables (interaction, self-regulation and self-efficacy) and satisfaction was identified in order to determine whether they were indicators of learners' satisfaction. The data were entered into SPSS 13.0 for processing and analyzing, whose results were shown in Table 1.

**Table 1:** **Results of data processing**.

**Scale**	**α**	**Spearman's rho**		**Descriptive statistics**											
				**Male**						**Female**					
		**Male**	**Female**	**M**	**SD**	**skewness**		**Kurtosis**		**M**	**SD**	**skewness**		**Kurtosis**	
				**S**	**S**	**S**	**SE**	**S**	**SE**	**S**	**S**	**S**	**SE**	**S**	**SE**
Interaction	0.82	0.88^**^	0.96^**^	2.94	0.73	0.37	0.23	−0.21	0.45	2.99	0.75	0.01	0.23	−1.16	0.45
Self-regulation	0.91	0.84^**^	0.98^**^	2.92	0.74	0.26	0.23	−0.72	0.45	2.98	0.74	0.03	0.23	−1.13	0.45
Self-efficacy	0.86	0.89^**^	0.94^**^	2.96	0.74	0.20	0.23	−0.77	0.45	2.97	0.73	0.04	0.23	−1.09	0.45
Satisfaction	0.92	1^**^	1^**^	2.91	0.71	0.13	0.23	−0.98	0.48	2.96	0.75	0.06	0.23	−1.19	0.45

** *Correlation is significant at the 0.01 level (2-tailed)*.

*M, means; SD, standard deviation; S, statistic; SE, Standard Error*.

As shown in Table 1, the first column showed the different scales; the second one displayed the Cronbach's alpha; the third two columns displayed Spearman's rho with satisfaction among males and females, followed by descriptive statistics. The Cronbach's alphas for interaction, self-regulation, self-efficacy and satisfaction were 0.82, 0.91, 0.86, and 0.92 respectively, all reaching satisfactory levels. All the scales used in the study were therefore considered internally consistent.

Furthermore, for males, interaction (ρ = 0.88, *p* <0.01), self-regulation (ρ = 0.84, *p* < 0.01), and self-efficacy (ρ = 0.89, *p* < 0.01) were positively correlated with satisfaction at the significance level 0.01. For females, interaction (ρ = 0.96, *p* < 0.01), self-regulation (ρ = 0.98, *p*< 0.01), and self-efficacy (ρ = 0.94, *p* < 0.01) were also positively correlated with satisfaction at the significance level 0.01. This meant, for both males and females, there was a positive relationship between interaction, self-regulation, self-efficacy and satisfaction. If participants interacted with peers or instructors more, or possessed stronger self-efficacy or self-regulation, they might feel more satisfied with learning aided with clickers, but more research was needed to establish a causal link. Anyway, there was a statistically significant relationship between interaction, self-regulation, self-efficacy, and satisfaction. It could be further inferred that interaction, self-regulation, and self-efficacy were indicators of satisfaction for both males and females. The first six established hypotheses, therefore, were all accepted.

### Structural equation modeling

**Figure 1:**
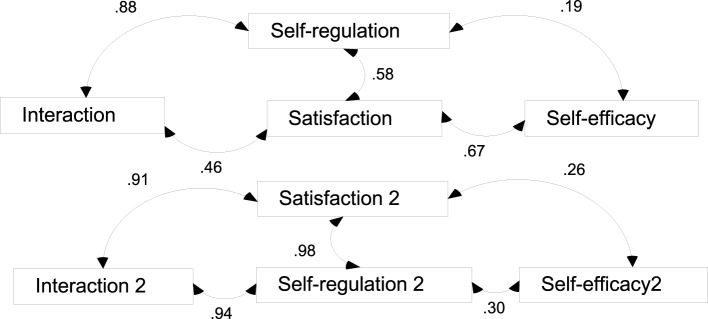
**Hypothesized model of the relationships between interaction, self-regulation, self-efficacy, and satisfaction**. Self-regulation, interaction, self-efficacy and satisfaction are measurements for males, while Self-regulation 2, interaction 2, self-efficacy 2, and satisfaction 2 are those for females. Chi-square = 480.351; Degrees of freedom = 18; Probability level = 0.000.

Before applying structural equation modeling (SEM), its assumptions were raised and investigated. The specifications of the model were for the mutual correlation between interaction, self-regulation, self-efficacy, and satisfaction for both genders. The result was summarized in Figure 1.

Figure 1 shows the results of the SEM analysis using maximum likelihood estimations. The path model showed that it is saturated, i.e., no unused degrees of freedom exist. Therefore, model is considered perfectly fit (Hu and Bentler, 1999). The standardized coefficients in Figure 1 demonstrate that self-regulation, interaction, self-efficacy, and satisfaction are positively related for both males and females.

There may be direct links between self-regulation and satisfaction. Students who are already of more self-regulation will have higher degrees of interactivity and self efficacy, and there will in turn be higher degrees of satisfaction. There may be causal link between self-efficacy and satisfaction, and students who are of more self-efficacy will be more self-regulated and interactive, and also more satisfied. Moreover, a causal link between interaction and satisfaction may also exist. Those who are more interactive may also be endowed with more self-regulation and self-efficacy, coupled with more satisfaction. Consequently, the hypothesis “self-regulation, interaction, self-efficacy and satisfaction are mutually correlated” is accepted.

## Discussion

It is unsurprising that interaction positively correlates with satisfaction. The interaction promoted by use of clickers between peers and the lecturer might have formed satisfaction among participants. It was argued (Smith, 1977; McKeachie, 1990) that participation and peer discussion could result in positively interactive learning activities. Clickers provide students with an effective way to encourage them to participate in classroom activities and peer discussion. It is commonly acknowledged that sharing and discussing viewpoints and issues among peers enable students to develop mutual understanding and cooperating in learning. Additionally, the process where students poll and then view the whole class polling, followed by communication with each other and discussion on interesting topics and difficult questions, provides students with a favorable opportunity of sharpening thinking and deepening understanding since they are exposed to a sea of both complementary and opposing opinions (Chickering and Gamson, 1991). New knowledge is therefore included into existing knowledge framework. In addition to use of clickers, the teachers designed other learning activities to make the class dynamic. Therefore, interaction might also have been promoted by other things except use of clickers.

My results suggest that lecturers should play a less active role in the classroom than commonly indicated (Fassinger, 1995). Instead, peer interaction among students should play an increasingly important role in learning and teaching process. It was supported by describing the way clickers were used in a developmental biology class to activate peer discussion on key conceptions in the course (Knight and Wood, 2005). Clicker questions were designed to measure students' understanding about the fundamental conception through individual response. The results showed that obvious disagreements significantly existed among students' understandings. Then the lecture grouped students into several subgroups to discuss the conceptions again. Students were also divided into subgroups to discuss general conceptions about developmental biology prior to the instructor's soliciting clicker responses. The findings indicated that in group discussion, students felt relaxed to meaningfully and actively debate the possible responses with their group members. This produced a generalized answer which was more reasonable than individual answer.

Cooperation among peers and increase in attendance facilitated by use of clickers might have enhanced participants' self-regulation, which possibly resulted in learner satisfaction. The powerful functions of clicker technology might be participation and discussion encouragement, coupled with attendance facilitation. Several studies showed that using a clicker system increased attendance. It was revealed (Homme et al., 2004) that students' attendance increased by 5%, which lasted for around 2 years. Another study reported that student attendance increased to 80–90% when clicker test scores formed the final academic result (Caldwell, 2007).

Cooperative learning aided with clickers among peers can be stimulated and promoted. Meanwhile, clickers could also encourage shy students to voice their opinions without risking being mocked at because shy students prefer to answer questions through anonymously clicking the button rather than raising their hands in public.

Satisfaction might also be resulted in because of self-efficacy in EFL (English as a foreign language) learning aided with clickers. Clickers might also enable shy students to become more self-confident and thus self-efficacy might be empowered. Shyness leads to students' drawing back from participation in classroom activities, which will exert negative influence on their academic performance. Young learners who were considered shy performed more poorly in vocabulary test than those who were not shy. Nevertheless, the difference in performance between shy and non-shy learners was not found when both shy and non-shy learners took the written test in groups where they could form peer discussion and poll anonymously compared with when they received the written test alone in the presence of invigilators (Crozier and Hostettler, 2003).

Several reasons why shyness could hinder student performance were explored (Mehrabian and Stefl, 1995). In the first place, responses realized by traditional hand-raising might be of less variety compared with keypad response. Keypad response might be able to enhance greater conformity to major opinions. Shyness might be eliminated when the opinion conformed to others. In the second place, clicker technology might enlarge the differences between shy and non-shy students, prompting shy students to be more subject to peer opinions. In the third place, anonymity attracted shy students more than non-shy ones because shy students would most likely feel easy in anonymous settings. Therefore, they might be more nervous when answering questions in public in class. Clicker technology equipped with anonymous polling function might have compensated for this regret.

Immediate feedback might enable participants to be more self-regulated, establishing satisfaction in learning. It was reported that both faculty and students at the University of Wisconsin believed the immediate feedback produced by the clicker technology was beneficial to the process of learning (Kaleta and Joosten, 2007). Faculty especially favored clickers due to the function of immediate feedback because it enabled them to evaluate students' understanding immediately. If the feedback indicated that students commanded the conception perfectly, then faculty could continue to the next topic, needless to worry about students' lagging behind. If the feedback showed that students did not understand the teaching contents to some degree, then faculty could slow down the lecturing or repeat some necessary contents. Faculty could also balance the lecturing speed and difficulties according to feedbacks. Students also reported the effectiveness of immediate feedback. Seventy-five percent students (*n* = 2013) reported that immediate feedback could enhance their knowledge and deepen their understanding about the course contents (Salmon and Stahl, 2005).

Participants might have felt satisfied with the use of clickers due to an essential function: anonymous polling. With anonymous polling, participants avoided the awkward situation when they made silly mistakes since peers could not spot out the mistake makers. Participants might be totally relaxed when responding to questions, which might also activate the participation in classroom activities. In addition, clickers might have provided participants with a wonderful platform to evaluate their own polling based on the histograms projected onto the large screen. In case they responded correctly, they might be satisfied with the result. When they found they made silly mistakes, they could discuss with their peers and tried to avoid them next time. In this way, mistakes gradually diminished and satisfaction grew up subconsciously.

## Conclusion

This study, by using bivariate correlation analysis, SEM and survey scales proved both valid and internally consistent, identified the indicators of satisfaction: interaction, self-regulation, and self-efficacy. This should be helpful to future researchers who desire to determine whether users are satisfied with the EFL teaching technology, as well as EFL teaching style and contents. This study could also set up a reference for instructors who desire to investigate the degree of satisfaction in the courses other than EFL.

Use of polling technologies such as clickers might satisfy learners since they could interact with peers through discussion, and possess self-efficacy and self-regulation through anonymous polling. Instant polling after peer discussion could encourage students to participate in learning activities without being nervous since the polling is anonymous. Proper teaching progress based on polling results could also content learners since most of them could keep pace with the progress. Better academic achievements through clickers-aided teaching, coupled with positive learning psychology, could also satisfy learners.

Nevertheless, clickers-aided EFL class would not be effective and efficient unless EFL teachers appropriately integrate polling technologies in their classrooms. Applying polling technologies to attendance check, EFL teachers could review what students have commanded, determine teaching progress based on students' mastery of language, and make EFL class dynamic through peer discussion. Clicker questions are essential to activate the class. They should help students master the linguistic knowledge such as vocabulary, sentence structure, and text perception. Prior to class, EFL teachers should also fully prepare for the clicker questions, the activity organization, and the potentially difficult problems.

There might be some defects in this study. Although there was evidence of construct validity and reliability in this study, the measurement of validity and reliability were conducted mostly in the context of western countries rather than in China. This study was conducted in China, and lecturers and participants were both from China. Different cultures might have exerted some influences on the validity and reliability, and the potential conflicts might, therefore, be unintelligible to outsiders. Another problem might be that participants from different majors were taught by different lecturers who might leave various teaching effects due to their different teaching styles. The gender of the teachers who might be biased toward use of technology might also play an important role in teaching and learning. Many factors other than clickers might have accounted for the between-class variation in the use of clickers. Different classes might differ in the familiarity with clickers, quality of clicker questions, the frequency with which clickers were used, and the nature of the subject matter. Considering the findings, one survey might not be enough to completely and convincingly determine the degree or the existence of satisfaction in EFL learning. Future studies on indicators of satisfaction could hardly succeed without cooperation from psychology, neurology, or any other related disciplines.

Clicker technology, despite its relatively new appearance and application in the classroom in higher education, continues the positive influence on learning and teaching. Numerous studies have obtained data indicating positive evaluation of clicker use among lecturers. However, students, as a main group of consumers, should act as important evaluators to measure the psychological acceptance or rejection toward or against clicker use. Consequently, the degree of psychological satisfaction among students, together with lecturers' assessment should be taken into a serious consideration. Previous literature, however, tended to study the satisfaction of clicker use in terms of involvement and performances, without considering students' inner psychological factors. It might be reasonable for future studies to shift from angles of student performances and lecturers' assessment to psychological measurement using experimental equipment in the field of psychology. How to judge whether users are satisfied with clicker technology in learning might never be an easy job which might need further cross-disciplinary cooperation from psychology, education, technology, and other related fields.

### Conflict of interest statement

The author declares that the research was conducted in the absence of any commercial or financial relationships that could be construed as a potential conflict of interest.
