# Supplementary material for: Indicators of satisfaction in clickers-aided EFL class
Source: Front Psychol. 2015 May 6;6:587. doi: 10.3389/fpsyg.2015.00587 (PMC4421939; doi:10.3389/fpsyg.2015.00587)
Supplement: Supplementary file 1 [file DataSheet1.DOCX]

**An appendix: scales to identify satisfaction, interaction, self-efficacy and self regulation**

**An interaction scale**

1. Clickers-aided EFL class provides a discussion platform for interaction.
2. Clickers-aided EFL class facilitates feedbacks from peers.
3. Clickers-aided EFL class presents an easy access to frequently asked questions.
4. Clickers-aided EFL class provides a place to discuss questions.
5. Generally, Clickers-aided EFL class contributes to the interactive capacity of students.

**A satisfaction scale**

1. Clickers-aided EFL class satisfactorily meets the information processing needs of the related area.
2. Clickers-aided EFL class is sufficient enough.
3. Clickers-aided EFL class is effective enough.
4. I am well satisfied with clickers-aided EFL class as a whole.
5. Clickers-aided EFL class helps improve the satisfaction of learning.
6. Clickers-aided EFL class helps in creating value for users.
7. Clickers-aided EFL class allows more learner orientation.

**A self-efficacy scale**

I feel confident:

1. Understanding terms/words relating to hardware of clickers.
2. Understanding terms/words relating to software of clickers.
3. Describing functions of clickers.
4. Trouble shooting problems of clickers.
5. Explaining why a task will not run through clickers.
6. Using the clickers to learn.
7. Learning advanced skills through clickers.
8. Turning to a peer discussion when needed.

**A self regulation scale**

1. If I study in appropriate ways, then I will be able to learn the material in this course.
2. It is my own fault if I don't learn the material in this course.
3. If I try hard enough, then I will understand the course material.
4. If I don't understand the course material, it is because I didn't try hard enough.

All the questions are followed by a five-Likert scale, i.e. *I strongly disagree, I disagree, I don't know, I agree, I strongly agree.*
